# Diversity and Metabolic Activity of Fungi Causing Biodeterioration of Canvas Paintings

**DOI:** 10.3390/jof8060589

**Published:** 2022-05-30

**Authors:** Cristina Lorena Văcar, Cristina Mircea, Marcel Pârvu, Dorina Podar

**Affiliations:** 1 Doctoral School of Integrative Biology, Babeș-Bolyai University, 400084 Cluj-Napoca, Romania; cristina.vacar@ubbcluj.ro; 2 Centre for Systems Biology, Biodiversity and Bioresources (3B), Babeș-Bolyai University, 3-5 Clinicilor St., 400084 Cluj-Napoca, Romania; cristina.mircea@ubbcluj.ro (C.M.); marcel.parvu@ubbcluj.ro (M.P.); 3 Department of Molecular Biology and Biotechnology, Babeș-Bolyai University, 1 M. Kogălniceanu St., 400084 Cluj-Napoca, Romania; 4 Department of Taxonomy and Ecology, Babeș-Bolyai University, 44 Republicii St., 400015 Cluj-Napoca, Romania

**Keywords:** culturable fungi, biodeterioration, canvas painting, hydrolytic enzymes, pigment solubilisation

## Abstract

Research into the biodeteriorative potential of fungi can serve as an indicator of the condition of heritage items. Biodeterioration of canvas paintings as a result of fungal metabolic activity is understudied with respect to both the species diversity and mechanisms involved. This study brings new evidence for the physiology of fungi biodeteriorative capacity of canvas paintings. Twenty-one fungal isolates were recovered from four oil paintings (The Art Museum, Cluj-Napoca) and one gouache painting (private collection), dating from the 18th to 20th centuries. The species, identified based on the molecular markers Internal Transcribed Spacer (ITS), beta-tubulin (tub2), or translation elongation factor 1 (TEF-1), are common colonisers of canvas paintings or indoor environments (e.g., *Penicillium* spp., *Aspergillus* spp., *Alternaria* spp.). Fungi enzymatic profiles were investigated by means of hydrolysable substrates, included in culture media or in test strips, containing components commonly used in canvas paintings. The pigment solubilisation capacity was assessed in culture media for the primary pigments and studied in relation to the organic acid secretion. Caseinases, amylases, gelatinases, acid phosphatase, N-acetyl-β-glucosaminidase, naphthol-AS-BI-phosphohydrolase, and β-glucosidase were found to be the enzymes most likely involved in the processes of substrate colonisation and breakdown of its components. *Aureobasidium* genus was found to hold the strongest biodeteriorative potential, followed by *Cladosporium*, *Penicillium*, *Trichoderma*, and *Aspergillus*. Blue pigment solubilisation was detected, occurring as a result of organic acids secretion. Distinct clusters were delineated considering the metabolic activities detected, indicating that fungi specialise in utilisation of certain types of substrates. It was found that both aged and modern artworks are at risk of fungal biodeterioration, due to the enzymatic activities’ diversity and intensity, pigment solubilisation capacity or pigment secretion.

## 1. Introduction

Canvas paintings, as cultural heritage artworks, represent invaluable assets for society and therefore require conscientious management to secure a perpetual legacy. Often, improper display or storage conditions, inadequate maintenance, or even human interventions can expose the integrity of the paintings, causing permanent damage. Biodeterioration is an adverse phenomenon resulting in mechanical, chemical or aesthetical destruction of materials as a consequence of microorganisms’ activity. The optimum conditions for the initiation of biodeterioration vary based on the microbial species that might be involved in the process; however, commonly, a relatively high humidity, temperature ranges from 20 to 30 °C, and substrate availability favour microorganisms’ colonisation of paintings [1,2]. Indoor climate, cleaning intervals, thermal insulation of the building, visitor flux, air streams, sunlight warming, and daily or seasonal changes in temperature gradients might also influence the progress of the biodeterioration process [3].

Chemically, canvas paintings are complex, consisting of various organic and inorganic substances. The organic materials found in oil canvas painting are commonly textile fibres in the support material, and glues, gelatine, casein, egg yolk, flour, rubber, oil, or resin, in the ground, pictorial, and protective layers [1,4]. Distinctively, paintings in gouache contain vegetal gums, dextrin or acrylic as binders [5]. Biologically, these compounds represent nutritive substrates for various types of microorganisms inhabiting canvas paintings that are therefore prone to undergo damage. The pigments usually consist of inorganic compounds, often heavy metal compounds, that may inhibit growth of certain microorganisms (both fungi and bacteria), due to toxic effects [6].

Airborne or human-inhabiting microorganisms represent an active component of painting deterioration, as under optimum conditions they may colonise the surface or layers of the paintings and thrive using its constituents. Unlike bacteria, microscopic fungi are able to use condensation water for growth [7], a process that is frequently associated with the deterioration of paintings stored in conditions where warm, humid air enters into contact with a cold surface. In addition, due to their filamentous growth, some fungal species are able to penetrate the painting structures using growing hyphae, which weakens the cohesion of the constituent layers, promoting thus exfoliations, cracking, and even loss of paint [1].

Prevention against biodeterioration, by indoor climate control, frequent cleaning and microbiological monitoring programmes, has only been practiced in important European museums for a few decades [3]. The impact of biodeterioration on numerous paintings belonging to collections that are less popular, but of inestimable value, remains unknown. The conventional disinfection procedures in art heritage restoration are limited in microorganisms’ inactivation efficacy, artwork integrity, personnel health and environmental safety [8]. For example, the gamma radiation doses used to achieve the killing of fungi are higher than those applied to bacteria, usually exceeding 10–20 KGy, and might alter the cellulose fibres [3,9]. Moreover, when evaluating or monitoring activities are conducted, assessing the diversity and the viability of microorganisms inhabiting heritage items is insufficient. Complementary studies of physiological activities are required for a better insight into the biodeterioration process, and to facilitate the development of customised treatments [10].

The diversity of microorganisms in relation to the biodeterioration of heritage items is well-documented for stone, wood objects, or mural paintings [11,12,13,14]. The biodeterioration of frescoes was addressed by consistent research, as wall paintings are more susceptible to microorganism attacks than easel paintings [15]. However, only a few studies have dealt with microbial communities inhabiting canvas paintings, especially the fungal ones [16,17,18,19]. Representatives of *Aspergillus, Penicillium, Cladosporium, Alternaria, Bacillus* are commonly found on historical canvas paintings, but the metabolites involved in the process are rarely examined [15,18,20,21]. The culturable fraction of the microbial diversity was successfully investigated for bacterial representatives (e.g., *Microbacterium*, *Bacillus*, *Sporosarcina*, *Paenibacillus*, *Reyranella, Staphylococcus*, *Acinetobacter*, *Agrococcus*) and their biodeteriorative potential was frequently associated with the activity of endocellulases, esterases, esterase lipases, phospholipases, or proteases [17,20,21,22]. Still, the fungal fraction is rarely recovered, and if it is successfully isolated, results in few representatives, low diversity, and limited study of their metabolic activity [16,17].

The aim of this research was to unveil the biodeteriorative potential of fungal communities inhabiting canvas paintings. Culturable fungi were isolated from paintings in oil and gouache, and molecularly identified. An enzymatic spectrum of the isolated fungi was established for substrates commonly used in canvas paintings. The pigment-solubilisation capacity was also assessed in vitro. Finally, principal component and hierarchical clustering analyses were used to detect any taxonomic patterns in the enzymatic profile.

## 2. Materials and Methods

### 2.1. Isolation and Identification of Culturable Fungi

The studied canvas paintings belong to the repository at The Art Museum of Cluj Napoca, i.e., Countess B. Mikes Istvánné b. Dujardin (anonymous, oil on canvas, 70 cm × 55 cm, 19th century, MA3355), Saint Matthew the Evangelist and the Angel (the manner of Caravaggio Michelangelo, oil on canvas 74 cm × 62 cm, 18th century, MA2605), Anny portrait (Nemeș Sabin, oil on canvas, 53 cm × 30 cm, 1961, MA8592), Mountain landscape (Gerhardt P. Alajos, oil on canvas, 49.5 cm × 64 cm, 19th century, MAFD261), and to a personal collection, namely the MD91 work, Marchel Ducharme, gouache on canvas, 55.5 cm × 71 cm, 1991. Each painting investigated throughout this study presented visible alterations, such as frame abrasions, scratches, craquelure, dust, or stains (Figures S1–S5). In terms of conservation state, the MA335 and MA2605 paintings were classified as “deteriorated”, while MA8592 and MAFD261 as “mediocre”. The MA3355 oil on canvas work presented evidence of a probably previous microorganisms’ attack on the reverse side, as white and grey concentric stains could be observed on the lower half. The MA2605 oil on canvas work presented small, circular, white accretions and signs of previous consolidation operations on the obverse side, while on the reverse, heavy dust particles and water stains could be observed. The MA8592 oil on canvas painting presented technical cracks and associated deformations on the obverse side, and also signs of infection, as a dark stain in the right upper corner of the reverse side. The MAFD261 oil on canvas painting, mostly altered by insufficient tension on the frame, that resulted in specific deformations and in cracks network, presented numerous black spots and colour alterations on the light-blue area on the observe side, and heavy dust particles on the reverse side. The MD91 gouache on canvas painting presented abundant brown concentric stains, with trails orientated downwards, on the reverse side.

Sampling was carried out non-invasively under aseptic conditions by gently rubbing sterile nitrocellulose membrane filters (Macherey-Nagel, Düren, Germany) and cotton paired swabs (VWR, Leicestershire, England) over the front and reverse areas visibly affected (for details, please see Figures S1–S5), then stored in sterile tubes for transportation to the laboratory. For each painting, the samples consisted of one nitrocellulose membrane and one cotton swab for the obverse side, and the same for the reverse side. The visibly affected areas were targeted for sampling to increase the chances of isolation of biodeteriorating fungi. The membranes and swabs were halved, one replicate was used for total DNA extraction, in order to characterise the fungal community by a metabarcoding approach, while the other replicate was used to recover culturable fungi. As the total DNA extraction with commercial kits (Quick-DNA Fecal/Soil Kit, Quick-DNA Fungal/Bacterial Kit, ZR Soil Microbe DNA, Zymo Research, Irvine, CA, USA) failed to pass the quantitative prerequisite for metabarcoding sequencing, further analysis was conducted on the culturable fungal community. Low DNA recovery is encountered for non-invasive samples of art objects [23], probably attributable to the presence of spores, which are more problematic for DNA extraction than mycelia [18]. Nevertheless, the recovery rate for fungi is assumed to be higher than 70% by cultivation techniques [3].

Both supports, filters and swabs used for fungi recovery, were inoculated directly onto Czapek-Dox agar (Formedium, Hunstanton, UK) and Sabouraud agar media (Hare, 2013), and the plates were incubated at 28 °C, for 7 days. Subsequently, colonies displaying distinct morphologies were selected for subculturing on Czapek-Dox agar plates, to obtain pure cultures. Stocks for storage at −80 °C were prepared in Czapek-Dox with 30% glycerol for each isolate. Seven-day-old cultures were used for the extraction of genomic DNA with Animal and Fungi DNA preparation kit (Jena Bioscience, Jena, Germany). The extracted DNA was used as template to amplify the internal transcribed spacer (ITS), translation elongation factor-1 (TEF-1), or tubulin beta chain (tub2) regions with MyTaq^TM^ Red Mix (Bioline, London, UK). The sequences of the primers used for amplification and their references are found in Table S1 [24,25,26,27]. The PCR products were purified with NucleoSpin Gel and PCR Clean-up kit (Macherey-Nagel, Düren, Germany) and sequenced by Macrogen (Amsterdam, The Netherlands). The amplified sequences were curated in Chromas (Version 2.6.6, 2018, Technelysium Pty Ltd., South Brisbane, Queensland, Australia), and used as queries in the BLASTn, NCBI databases. Species of the entries with the highest total score were assigned to the inquired sequence. The sequences of identified fungal strains were deposited under ON359960–ON359969 (ITS), ON491577–ON491582 (Bt2), and ON491583–ON491586 (EF1) accession numbers.

### 2.2. Enzymatic Profile Characterisation

The biodeteriorative potential of the isolated fungi was assayed for six classes of enzymes: lipases, esterases, lecithinases, caseinases, gelatinases, and amylases. These enzymes are able to hydrolyse substrates that are commonly used in the preparation of historic canvas paintings, i.e., vegetable oils, egg yolk, milk casein, animal glues or flour. Lipase and esterase activities were assessed in Tween substrate plates, containing 10 g/L peptone, 5 g/L NaCl, 0.1 g CaCl_2_ × H_2_O, 15 g/L agar and 10 mL Tween 80 or Tween 20, respectively, pH 7.4 [28]. Lecithinase activity was evaluated in egg yolk agar plates, containing 40 g/L tryptone, 5 g/L Na_2_HPO_4_, 2 g/L NaCl, 0.01 g/L MgSO_4_ × 6 H_2_O, 2 g/L glucose, 15 g/L agar, supplemented with 10% egg yolk emulsion, pH 7.6 [29]. Caseinase activity was examined in media containing 28 g/L skim milk powder, 5 g/L tryptone, 2.5 g/L yeast extract, 1 g/L glucose, and 15 g/L agar, pH 7.5 [30]. Gelatinase activity was assessed in media containing 5 g/L digest of gelatine, 3 g/L beef extract, and 120 g/L gelatine, pH 7.5 [31], dispensed in test tubes. Amylase activity was evaluated with the starch agar protocol [32]. The plates were inoculated with a loop of mycelia from seven-day old cultures in two spots, while test tubes for the gelatinase test were centrally inoculated. The plates were incubated for 7 days at 28 °C, and the test tubes at 22 °C. The cultures were inspected at three and six days of incubation. To evaluate the gelatinase activity, the test tubes were kept for 1h at 4 °C before inspection. The intensity of the enzymatic activity was scored on a scale from 0—no enzymatic activity, 1—low enzymatic activity, 2—medium enzymatic activity, to 3—maximum enzymatic activity observed. To compliment these observations, the enzymatic activity of 19 hydrolytic enzymes was assessed semi-quantitatively with the API ZYM test strips (#25200, bioMérieux, Marcy-l’Etoile, France), as previously proved convenient for characterisation of the enzymatic profile of bacteria inhabiting canvas paintings [17,18]. The intensity of the reaction was scored as 0, 1, 2, 3, 4 and 5, representing 0, 5, 10, 20, 30, and >40 nM hydrolysed substrate, respectively, according to the manufacturer instructions. Scores ≥ 3, equivalent to more than 20 nM hydrolyses substrate were considered positive for the API ZYM test strips, as indicated by the producer.

### 2.3. Pigment Solubilisation Capacity

The pigment solubilisation capacity was assessed according to Pavić et al. [22]. The modified metal toxicity medium (mMT) was prepared by dissolving 10 g/L glucose, 2.13 g/L Na_2_SO_4_, 0.06 g/L CaCl_2_, 1 g/L NH_4_Cl, 1 g/L MgSO_4_, 0.05 g/L yeast extract, 0.5 g/L tryptone, 15 g/L agar and 1 g/L pigment powder in distilled water, pH 7, followed by autoclaving. The pigments were selected to include the primary colours used in paintings; red, yellow, and blue, and also white and black. The pigments were red-PR102 (Fe_2_O_3_), yellow-PY3 (arylamide), blue-PB29 (2SNa_2_ × 3Na_2_O × 3Al_2_O_3_ × 6SiO_2_), white-PW6 (TiO_2_), and black-PBk11 (Fe_3_O_4_, traces of SiO_2_ and Al_2_O_3_), purchased from Divolo Firenze (Montepulciano Stazione, Italy). The plates were inoculated with a loop of mycelia from seven-day old cultures in duplicate, and incubated at 28 °C in darkness. After 7 days of incubation, the growth and solubilisation diameters were measured, and any visual alterations of the media and the mycelia were observed. In order to assess whether the solubilisation of pigments is the result of organic acid secretion, bromothymol blue was added to the mMT as a pH indicator [33].

### 2.4. Statistical Analyses

One-way ANOVA followed by Tukey’s multiple comparisons test was performed using Minitab^®^ version 17.1.0 for Windows [34], to discern among the enzymatic activities potentially involved in biodeterioration. Principal Component Analysis (PCA) was employed to examine taxonomic patterns in the enzymatic profile, using mean intensity scores per genera in Past 4.03 software [35]. The matrix plot representation was developed in Past 4.03 software. Hierarchical Clustering based on dissimilarities, for the enzymatic activities coupled with organic acid secretion, was employed to feature eventually specialised clusters [36]. For the Hierarchical Clustering Analysis, the API scores were converted to correspond with the scores obtained for hydrolysable substrates in culture (i.e., 0–2 converted to 0, 3 to 1, 4 to 2, and 5 to 3), while for the organic acid secretion, a solubilisation index (S.I.) was generated by dividing the diameter of the solubilisation zone to the hypothallus diameter, and this was fitted on the same scale.

## 3. Results

### 3.1. Diversity of Culturable Fungi

**Figure 1:**
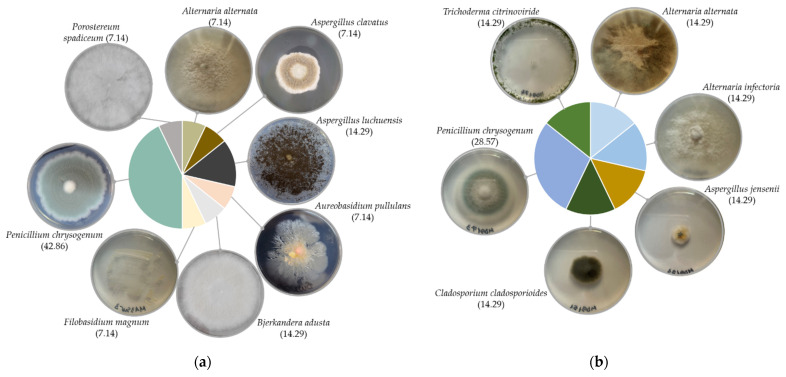
Fungi diversity and colony morphology, on Czapek-Dox medium, of fungi isolated from: (**a**) oil on canvas paintings (MA3355, MA2605, MA8592, MAFD261); (**b**) gouache on canvas painting (MD91); percentages of species are indicated between the brackets.

Twenty-one fungal isolates were recovered in total; 11 distinct species belonging to eight genera, mostly from the back side of the paintings. Fungi from the front side were isolated only from the MD91 painting, in gouache technique. Recovered fungi belonged to Ascomycota (86%) and Basidiomycota (14%) phyla. Isolates belonging to the *Penicillium* genus were the most abundant, followed by *Aspergillus*, and *Alternaria*, in both techniques’ paintings. Members of the genera *Aureobasidium*, *Bjerkandera*, *Cladosporium*, *Filobasidium*, *Porostereum* and *Trichoderma*, were occasionally isolated. The oil paintings harboured members of both Ascomycota, mostly *Penicillium chrysogenum*, followed by *Aspergillus* spp., and occasionally *Alternaria alternata*, *Aureobasidium pullulans*, and Basidiomycota, with one isolate for *Bjerkandera adusta*, one for *Filobasidium magnum*, and one for *Porostereum spadiceum* (Figure 1a). Only Ascomycota representatives were recovered from the gouache painting, i.e., *Alternaria* spp., *Aspergillus jensenii*, *Cladosporium cladosporioides*, *Penicillium chrysogenum*, and *Trichoderma citrinoviride* (Figure 1b).

### 3.2. Enzymatic Profile Characterisation

**Figure 2:**
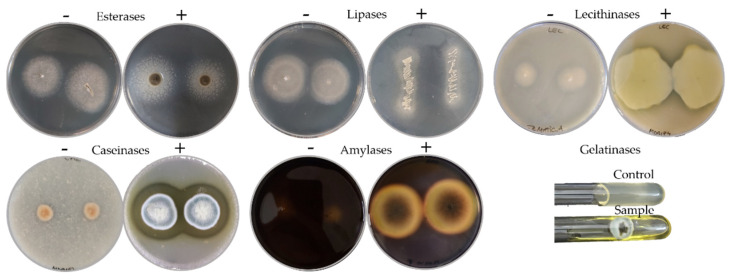
Representative positive plates (+) or test tubes of fungal isolates for the hydrolytic enzymes assayed in comparison with negative plates (−).

The biodeteriorative potential of the fungal isolates was established based on their enzymatic profile in specific culture media agar, i.e., the presence and the intensity of the enzymatic activity for esterases, lipases, lecithinases (lipolytic enzymes), caseinases, gelatinases (proteolytic enzymes), and for amylases (starch hydrolytic enzymes) (Figure 2).

**Figure 3:**
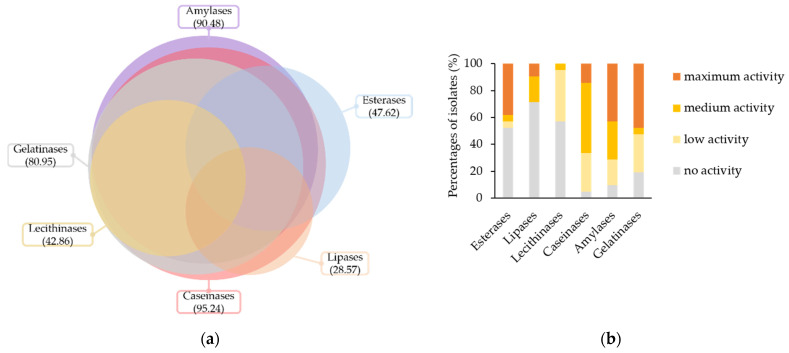

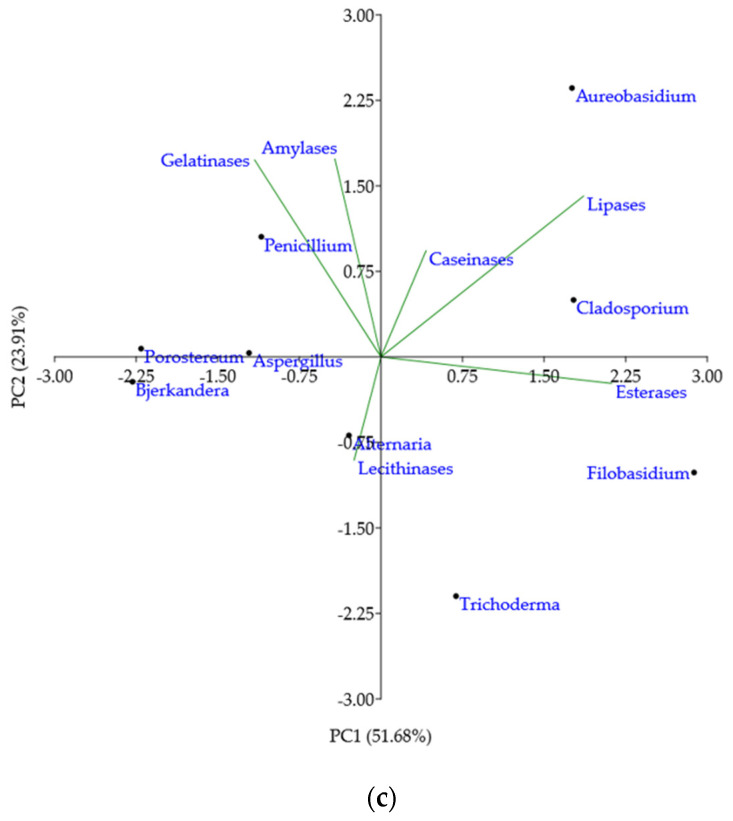
Biodeteriorative enzymatic profile of isolated fungi according to substrate hydrolyzation in culture: (**a**) Occurrence of hydrolytic enzyme activities in the culturable fungal population, expressed as percentages; (**b**) percentages of isolates according to enzymes’ activity intensity scores; (**c**) principal component (PC) analysis biplot, for the first two principal component scores, using mean enzymatic activities intensities per fungal genera.

An overview of the biodeteriorative enzymatic potential of the isolated fungi is presented in Figure 3. Most of the isolates, i.e., 95.24%, were positive for the activity of caseinases, 90.48% of the amylases, 80.95% of the gelatinases, 47.62% of the esterases, 42.86% of the lecithinases, and 28.57% of the lipases (Figure 3a).

Based on the intensity of enzymatic activities in culture (Figure 3b), 47.62%, 42.86%, 38.1%, 14.29% and 9.52% of isolates had maximum activities for gelatinases, amylases, esterases, caseinases and lipases, respectively. Intermediate activities, i.e., low and medium scores, were recorded for 80.95%, 47.62%, 42.86%, 33.33%, 19.05%, and 9.52% of isolates in the presence of substrates for caseinases, amylases, lecithinases, gelatinases, lipases and esterases, respectively.

Mean of the enzymatic activities intensities in culture per genera were analysed by PCA to outline taxonomic trends with respect to the enzymatic profile (Figure 3c). According to the loading weights, PC1 was dominated by the difference between (esterases + lipases) and gelatinases, while PC2 was dominated by the sum of lipases, amylases and gelatinases. Caseinases and lecithinases justified the least variance of the data. Lecithinases were negatively correlated with caseinases and lipases, while caseinases were uncorrelated with gelatinases, amylases, and esterases. The PCA scores indicated that *Aureobasidium* and *Cladosporium* genera had broad and intense enzymatic activities for the substrates assayed, except for lecithin. *Filobasidium* genus had a large PC1 value, denoting strong esterases and lipases activity, but no activity for other enzymes. *Trichoderma* genus position, on the bottom of the plot, implied medium activity for lecithinases, esterases, or caseinases, but no lipases, amylases or gelatinases activity. Extreme left position for *Bjerkandera* and *Porostereum* genera showed mostly low or no enzymatic activity, while *Alternaria* genus position indicated low or moderate broad enzymatic activity. *Aspergillus* and *Penicillium* genera plotting illustrated moderate to high gelatinases, amylases, and caseinases activities, but low esterase, lipase and lecithinase activities. Concerning the spectrum and the intensity of enzymatic activity, *Aureobasidium* genus holds the strongest biodeteriorative potential, followed by *Cladosporium*, *Penicillium*, *Trichoderma*, and *Aspergillus*.

**Figure 4:**
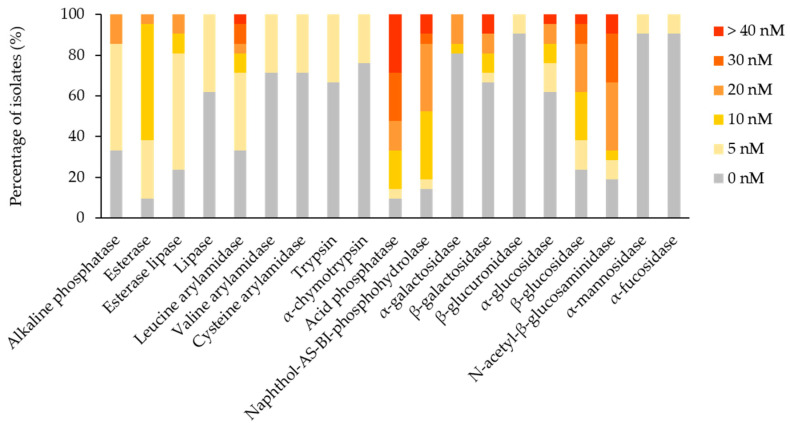
Percentages of fungal isolates displayed according to the substrates’ hydrolyzation intensities, expressed in nM, detected by the API ZYM test strips.

The enzymatic profile of each isolate was assessed for 19 enzymes by the API ZYM test strips. Considering the positive results, a reaction intensity score ≥3, 11 substrates were hydrolysed by the isolates. Acid phosphatase and N-acetyl-β-glucosaminidase were the most frequently detected enzymes (66.67% of isolates), followed by naphthol-AS-BI-phosphohydrolase (47.62%), β-glucosidase (38.1%), leucine arylamidase and β-galactosidase (19.05%), alkaline phosphatase, α-galactosidase and α-glucosidase (14.29%), esterase lipase (9.52%), and esterase (4.76%) (Figure 4). Negative results, a reaction intensity score < 3, were registered for lipase, valine arylamidase, cysteine arylamidase, trypsin, α-chymotrypsin, β-glucuronidase, α-mannosidase, and α-fucosidase. In general, the activity of enzymes falls in between the culture and the strip assays, i.e., esterases and lipases, corroborated. Considering the frequency and intensity of enzymatic activities among the isolated fungi, it was found that acid phosphatase, N-acetyl-β-glucosaminidase, naphthol-AS-BI-phosphohydrolase, and β-glucosidase (statistically significant difference for *p* < 0.0001) are likely potential agents promoting fungal colonisation of canvas paintings. In addition, esterases (statistically significant difference for *p* > 0.01) and leucine arylamidase (statistically significant difference for *p* > 0.05) might contribute to the biodeterioration process.

**Figure 5:**
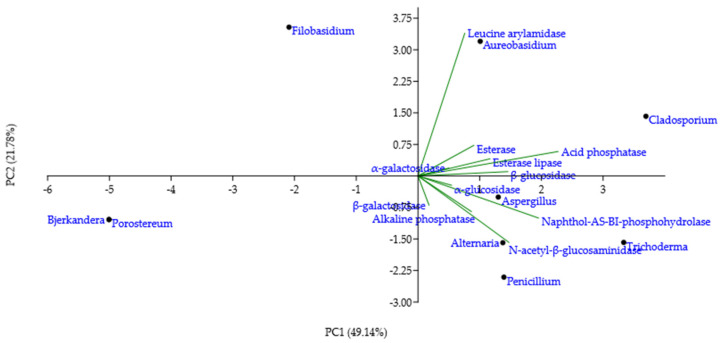
Principal component (PC) analysis biplot, for the first two principal component scores, using mean enzymatic activities intensities per fungal genera, as detected by the API ZYM test strips.

PCA was applied to the mean of the reaction intensity scores per genera, for the enzymes with a score higher than 0, to detect taxonomic patterns in the enzymatic profile detected by the test strips (Figure 5). The loading weights exposed the sum of acid phosphatase and naphthol-AS-BI-phosphohydrolase as dominant variables of PC1, while PC2 was influenced mostly by leucine arylamidase. Esterase, esterase-lipase, α-galactosidase, β-glucosidase, α-glucosidase, alkaline phosphatase, and β-galactosidase contributed less to the overall variation per genera variation. *Cladosporium* genus position on the plot highlighted a broad enzymatic activity, especially for leucine arylamidase, acid phosphatase, naphthol-AS-BI-phosphohydrolase and N-acetyl-β-glucosaminidase. *Trichoderma* genus had a high PC1 value, which is indicative of strong naphthol-AS-BI-phosphohydrolase activity. *Alternaria* and *Penicillium* genera plotting showed strong N-acetyl-β-glucosaminidase, in addition to a broader enzymatic spectrum. *Aspergillus* genus, situated in the centre of the plot, indicated moderate, broad enzymatic activity. *Bjerkandera* and *Porostereum* genera, located in the leftmost corner of the plot, illustrated a lack of enzymatic activity. *Filobasidium* and *Aureobasidium* genera, having a high PC2 value, demonstrated pronounced leucine arylamidase activity and a negligible activity for other enzymes.

### 3.3. Pigment Solubilisation Capacity

**Figure 6:**
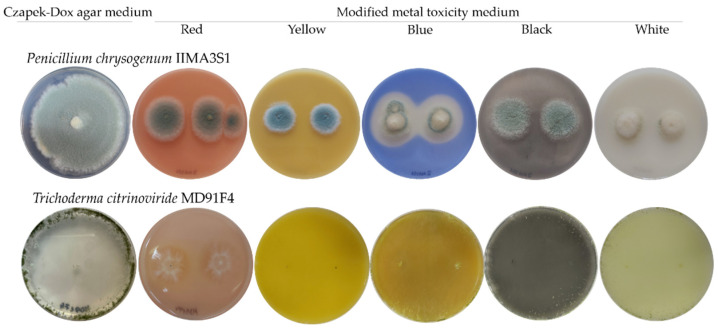
Alterations of the pigments induced by fungi on modified metal toxicity medium, individually supplemented with the primary pigments. Photos were taken at 7 days of incubation at 28 °C. *Penicillium chrysogenum* IIMA3S1 isolate, solubilises the blue pigment, and *Trichoderma citrinoviride* MD91F4 isolate, solubilises the blue pigment and induces colour alteration in the blue and white pigment-containing media. For reference, the cultures of these isolates are shown on Czapek-Dox agar medium.

Furthermore, the potential of the fungal isolates to deteriorate the canvas paintings by inducing any type of alterations to the pigments themselves was investigated. Thus, the fungal isolates were cultured on a pigment-containing medium to assess whether the main pigments used in the paintings could be solubilised by fungal colonisation (Figure 6). The blue pigment was the only one susceptible to fungal discolouration, i.e., 61.9% of isolates displayed solubilisation capacity of the blue pigment, as clear areas developed around the growing colonies. The species that were able to induce discolouration of the blue pigment were *Aspergillus clavatus, Aspergillus luchuensis, Aureobasidium pullulans, Bjerkandera adusta, Penicillium chrysogenum*, and *Trichoderma citrinoviride*. However, red, yellow, black and white pigments did not deteriorate. In addition, the blue, white, and red pigments in the media induced depigmentation in 62.5%, 50%, and 6.25%, respectively, of the pigmented fungal colonies. Colony depigmentation in the presence of the blue pigment occurred in the case of *Penicillium* genera, and of two isolates of the *Aspergillus* genera. The white pigment induced depigmentation in six *Penicillium*, one *Aspergillus*, and one *Alternaria* isolates. Another type of substrate alteration was observed for the *T. citrinoviride* MD91F4 isolate, which, in the presence of blue and white pigments modified the colour of the medium, inducing a yellow tint.

**Figure 7:**
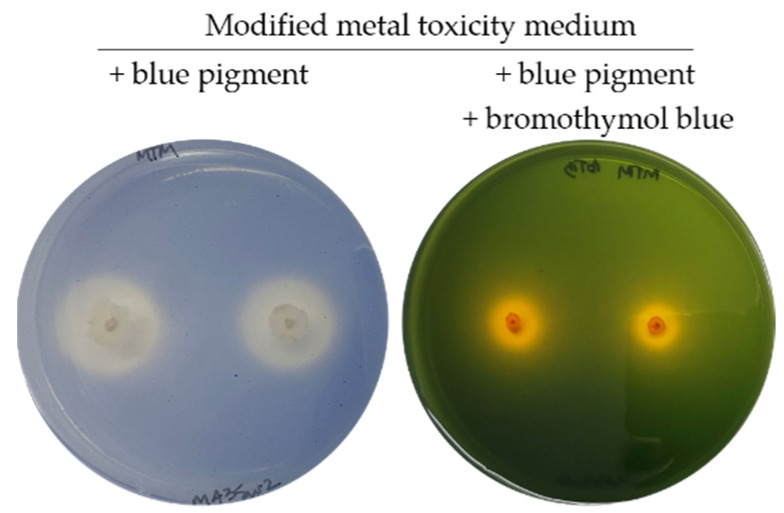
Fungi ability to solubilise the blue pigment, on a modified metal toxicity medium, is the result of organic acid secretion, as highlighted by the bromothymol blue addition to the blue pigment-containing medium.

As correlation statistics showed no relationship between the blue pigment solubilisation and the activity of any enzyme assayed, the possibility of solubilisation via organic acid secretion was examined by supplementing the pigment-containing medium with the pH indicator bromothymol blue (yellow at pH ≤ 6, green at 6 ≤ pH ≤ 7.6, and blue at pH ≥ 7.6). This approach validated that secreted organic acids were responsible for blue pigment solubilisation, as all tested colonies developed yellow halos on medium containing blue pigment and bromothymol blue, indicating that the pH of the medium dropped below 6, while the pH of the surrounding medium, uncolonised, ranged between 6 and 7.6 (Figure 7).

**Figure 8:**
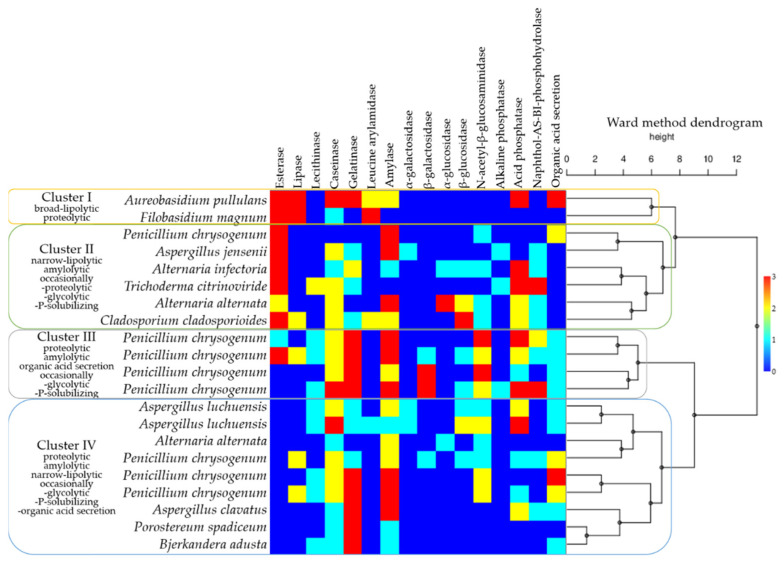
Biodeteriorative fungal clusters according to enzymatic and blue pigment solubilisation activities, based on a Ward method dendrogram coupled with a matrix plot representing activity intensities (scaling from blue—no activity, to red—maximum activity). Note that even when the species’ names are identical, they represent different isolates, independently obtained.

In order to facilitate a broader perspective on the biodeteriorative potential of the fungal isolates, the enzymatic activities detected in the culture and by API ZYM test strips were integrated with the organic acid secretion, responsible for the blue pigment solubilisation. A dendrogram was generated, based on the Ward method, and joined with the matrix plot representation of the intensity of enzymatic activities (Figure 8). Four clusters were delineated, based on similarities among the hydrolytic capacities. *Filobasidium magnum* (Basidiomycota) clustered with *Aureobasidium pullulans* (Ascomycota), due to broad lipolytic and proteolytic activities. The second cluster comprised of *Alternaria alternata*, *Alternaria infectoria, Aspergillus jensenii*, *Cladosporium cladosporioides*, *Trichoderma citrinoviride*, and a *Penicillium chrysogenum* isolate (all Ascomycota, originating from the gouache-painting), able to hydrolyse short-chain fatty acids, in addition to amylolytic, and occasionally proteolytic, glycolytic and P-solubilising activities. The third cluster, defined by proteolytic, amylolytic, organic acid secretion and occasionally glycolytic, and P-solubilising activities, included representatives of the *Penicillium*. The fourth cluster was represented by *Alternaria alternata*, *Aspergillus clavatus*, *Aspergillus luchuensis*, *Penicillium chrysogenum* (Ascomycota), *Bjerkandera adusta* and *Porostereum spadiceum* (Basidiomycota), which presented proteolytic, amylolytic, narrow-lipolytic (for lecithin), and occasionally glycolytic, P-solubilising and organic acid secretion activities.

## 4. Discussion

Understanding the process of biodeterioration is an integral part of the conservation process of the heritage artworks. Biodeterioration of frescoes was frequently studied, as they are more exposed to unfavourable conditions than canvas paintings, and therefore more prone to deterioration [1]. Nevertheless, canvas paintings, commonly exhibited or stored in indoor environments, are susceptible to biodeterioration too, due to their organic matter abundance. Important progress has been made in the field of canvas paintings biodeterioration, especially with respect to microbial species diversity [19,20,22,23,37]. The bacterial biodeteriorative enzymatic repertoire was occasionally examined [16,17,24]. However, fungal mechanisms remain elusive, as few studies attempted to characterise their enzymes involved in canvas paintings biodeterioration [21,38,39,40].

The current study was conducted on four oil on canvas paintings, from the repository of The Art Museum, Cluj-Napoca, and one gouache on canvas painting, from a personal collection. No evident mycelia could be observed on the surfaces of the studied paintings. However, the presence of dust, accretions and stains, especially on the observe side, indicated that the surfaces are colonised by fungi. Twenty-one fungal colonies were isolated by non-invasive techniques from the analysed artworks. The fungal diversity was dominated by *Penicillium*, followed by *Aspergillus* and *Alternaria* genera (Figure 1). Representatives of *Aureobasidium*, *Bjerkandera*, *Cladosporium*, *Filobasidium*, *Porostereum*, and *Trichoderma* genera were also isolated. Some of these species, i.e., *Alternaria* spp., *Aspergillus* spp., *Aureobasidium pullulans*, *Cladosporium* spp., *Penicillium* spp., are common colonisers of paintings [3]. The prevalence of *Penicillium* spp. on paintings was frequently reported [17,37], and the order of genera abundance is in accordance with previous studies [20,21,37]. Therefore, the fungal diversity detected in our current study is consistent with other indoor environments.

Ascomycota phylum dominates a wide range of habitats, including canvas paintings. In contrast, Basidiomycota representatives inhabiting canvas paintings were detected previously only by a metagenomic approach, accounting for 14.5% of the total fungal population [23]. We report the presence of Basidiomycota representatives, recovered by a culture-dependent method, i.e., *Filobasidium magnum* (Tremellomycetes class, Filobasidiales order), *Bjerkandera adusta* and *Porostereum spadiceum* (Agaromycetes class, Polyporales order). *F. magnum* has a wide range of habitats, from plant epiphytic, rhizosphere soil, to indoor habitats and even humans [41,42,43,44,45,46]. *F. magnum* was isolated with high frequency from bedding [44] and from wet household surfaces [45], suggesting that this species thrives in humid indoor environments. *B. adusta* is a macromycete species, naturally encountered as a saprotroph on dead hardwood, which proved efficient for the degradation of synthetic dyes [47,48,49,50]. Similarly, *P. spadiceum* colonises typically dead hardwood, and demonstrated potential for the biological treatment of landfill leachates and textile wastewaters [51,52,53]. Moreover, a large-scale study targeting indoor wood-decaying fungi in 2000 buildings over a 9-year period, identified *B. adusta* in beams and windows of houses [54]. However, most of the fungal species identified in the current study are also known to be associated with respiratory, eye and skin symptoms in indoor environments [55,56,57,58].

The biodeteriorative potential of *Aspergillus* spp. and *Penicillium* spp., originating from indoor air of cultural heritage conservation premises, was attributed to proteolytic, cellulolytic, acid production and secretion of extracellular pigments [59]. Mixed fungal-bacterial inocula were applied to mock layers, representative of the tempera painting under study, to find out that tempera paints were heavily biodegraded, and the plasticisers and glue were degraded to a certain extent, while the varnishes were the least affected [21]. However, the biodeteriorative potential of fungi is scarcely documented for canvas paintings. Fungal isolates recovered from biodeteriorated oil paintings on canvas commonly displayed esterase, N-acetyl-β-glucosaminidase, naphtol-AS-BI-phosphohydrolase, and acid phosphatase activities [17,18]. In addition to these enzymes, alkaline phosphatases, esterase lipases, leucine arylamidases, caseinases, gelatinases, amylases and β-glucosidases were frequently detected among isolates investigated under the current study. Considering the frequency and intensity of enzymatic activities among the isolated fungi, the enzymes caseinases, amylases and gelatinases (statistically significant difference for *p* < 0.05) represent the principal biodeteriorative enzymes that likely facilitate the colonisation of the canvas paintings. Accordingly, these fungal species are able to lyse especially components in the ground layers, i.e., milk casein, starch or animal glues. Esterases also contribute to the biodeteriorative repertoire, although lipases and lecithinases appear to have minor involvement in the biodeteriorative process. It was stated that esterases and lipases, which hydrolyse soluble and insoluble, respectively, lipids in paintings’ emulsifiers, are the main hydrolytic enzymes responsible for canvas biodeterioration [60]. The activity of N-acetyl-β-glucosaminidase, which allows hydrolysation of the polysaccharides in the bacterial cell wall peptidoglycan, is often reported for fungi involved in canvas paintings biodeterioration [17,18]. Phosphatases, i.e., naphthol-AS-BI-phosphohydrolases, acid and alkaline phosphatases, might be involved in phosphate release from pigments [22]. Leucine-arylamidases, caseinases, and gelatinases are probably involved in the degradation of glue in the ground layer, or of casein in binders, supplying the N source for fungal growth. The starch used in restoration interventions is prone to amylase action [61], and together with the activity of detected β-glucosidases, that convert short oligosaccharides to glucose [15], should satisfy the carbon demand of fungal populations. Concerning the canvas support degradation capacity, we did not have direct evidence for textile fibres degradation. However, due to detection of glycolytic enzymes activity and the presence of wood-decaying species, it is presumed that the canvas support is also prone to fungal biodeterioration. The overall spectrum of the enzymatic activities detected was similar between the two techniques investigated. Therefore, the current study revealed enzymes potentially involved in fungal biodeterioration that previously were not considered in relation to canvas paintings. In addition, the metabolic versatility and competitivity of fungal communities inhabiting canvas paintings were reinforced by this research.

Pigment biodeterioration was previously studied only in relation to prokaryotic representatives. Bacteria isolated from a canvas painting were able to solubilise Zinc white or Cobalt deep green in culture, probably due to acid-metal complexation, as a result of organic acids release during glucose metabolism [22]. Mock paintings, individually prepared with ultramarine blue or chrome yellow in casein binder and infected with proteolytic bacteria, developed structural and aesthetic alterations due to degradation of the binder layer [20]. However, the pigment degradation alone was not considered. The ability of fungi to interact with pigments used in paintings has not been previously shown. Herein, we report the capacity of fungal isolates recovered from canvas paintings to solubilise the blue pigment. The mechanism of blue pigment discolouration is likely due to secretion of organic acid, as bromothymol blue indicated a drop in the pH within the solubilisation areas. It is possible that the released organic acids complex Al atoms from the pigment, as the ultramarine blue colour loss was attributed to the removal of Al atoms from the pigment framework which allowed the release of the inner chromophore [62]. It is not clear why only the blue pigment suffered alterations as a result of fungal activity. The precise mechanism of the degradation of the primary pigments investigated has not yet been elucidated. It is possible that red-PR102 (Fe_2_O_3_), yellow-PY3 (arylamide), white-PW6 (TiO_2_), and black-PBk11 (Fe_3_O_4_, traces of SiO_2_ and Al_2_O_3_) pigments might be stable under acidic conditions due to their structural characteristics. However, further investigation is required to establish the causality between secreted organic acids and blue pigment discoloration. Other observations related to the interaction between isolated fungi and the primary pigments tested support previous findings that state the inhibitory effect of pigments against microorganisms’ populations in a wood painting [6]. The effects reported herein include the reduction in growth diameter and/or colony depigmentation in pigmented fungal isolates. In addition, the induction of a yellow tint in the pigmented culture media was observed for *T. citrinoviride* in the presence of blue and white pigments. It is likely that the effect was due to secreted cyclic polyketides sorbicillinoids having a characteristic yellow-orange colour and high antioxidant and radical scavenging activities [63,64]. Sorbicillinoids are a family of complex cyclic polyketides restricted to few distantly related ascomycetes. They are synthesised under conditions of rapid growth (glucose as carbon substrate) by a gene cluster subjected to lateral gene transfer (LGT) and loss of gene events within Fungi [64]. It is inquiring that yellow pigmentation of the medium does not occur in the absence of the pigments, despite containing the same carbon source. However, the presumed secretion of sorbicillinoids might act as a defence strategy against toxicity of pigments. The medium containing red pigment did not suffer evident alterations, probably due to its toxic effect that inhibited *T. citrinoviride* growth. It is unclear why the pigmentation of the surrounding medium occurs only in the case of *T. citrinoviride* as other ascomycetes isolated within this study are known as pigment producers, and were subjected to the same conditions. It is possible that either the lack of LGT or loss of gene events in these isolates may result in limited secondary metabolites production. Otherwise, the isolates may require specific conditions for production of pigments that results in substrate tinting.

## 5. Conclusions

Considering the vulnerability of canvas paintings, both ancient and modern, facing the hazard of biodeterioration, there is growing interest in understanding this process to enable the development of suitable conservation techniques and practices. The current research provides an insight into the culturable diversity and metabolic biodeteriorative potential of fungal communities inhabiting canvas paintings. The composition of fungal population was typical to those found on cultural heritage objects, and those found in indoor environments. Representatives of *Penicillium*, *Aspergillus*, and *Alternaria* were frequently isolated, and *Aureobasidium*, *Bjerkandera*, *Cladosporium*, *Filobasidium*, *Porostereum* and *Trichoderma*, were occasionally isolated. The biodeteriorative potential indicated a high risk for the studied artwork, both in terms of enzymatic diversity and activity intensity, mostly responsible for the deterioration of the supports and the ground layers. Enzymes most likely responsible for biodeterioration were caseinases, amylases, gelatinases, acid phosphatase, N-acetyl-β-glucosaminidase, naphthol-AS-BI-phosphohydrolase, and β-glucosidase, occasionally complimented to a lesser extent by esterases, lipases, lecithinases, and leucine arylamidase. In addition, discolouration of the blue pigment was demonstrated, likely as a result of metal complexation to organic acids secreted.

## Data Availability

Not applicable.

## Supplementary Materials

The following supporting information can be downloaded at: https://www.mdpi.com/article/10.3390/jof8060589/s1, Table S1: Primers used for fungal identification; Table S2: Identity of the isolated fungal species according to the blastn results of the amplified molecular markers. Figure S1: Oil on canvas painting MA3355, in the repository of The Art Museum, Cluj-Napoca, Romania, with red rectangles indicating the sampling areas and fungal isolates inhabiting the reverse of the support; Figure S2: Oil on canvas painting MA2605, in the repository of The Art Museum, Cluj-Napoca, Romania, with red rectangles indicating the sampling areas and fungal isolates inhabiting the reverse of the support; Figure S3: Oil on canvas painting MA8592, in the repository of The Art Museum, Cluj-Napoca, Romania, with red rectangles indicating the sampling areas and fungal isolates inhabiting the reverse of the support; Figure S4: Oil on canvas painting MAFD261, in the repository of The Art Museum, Cluj-Napoca, Romania, with red rectangles indicating the sampling areas and fungal isolates inhabiting the reverse of the support; Figure S5: Gouache on canvas painting MD91, from a personal collection, Cluj-Napoca, Romania, with red rectangles indicating the sampling areas and fungal isolates inhabiting the obverse (on the left) and the reverse (on the right) of the support.
