# Supplementary material for: Diversity and Metabolic Activity of Fungi Causing Biodeterioration of Canvas Paintings
Source: J Fungi (Basel). 2022 May 30;8(6):589. doi: 10.3390/jof8060589 (PMC9224695; doi:10.3390/jof8060589)
Supplement: Supplementary file 1 [file jof-08-00589-s001.zip › jof-1733383-supplementary 20220531.pdf]

Table S1. Primers used for fungal identification.

Primer	Sequence (5′-3′)	Reference
ITS1F	CTTGGTCATTTAGAGGAAGTAA	[24]
ITS4	TCCTCCGCTTATTGATATGC	[25]
EF1-1018F	GAYTTCATCAAGAACATGAT	[26]
EF1-1620R	GACGTTGAADCCRACRTTGTC	
Bt2a	GGTAACCAAATCGGTGCTGCTTTC	[27]
Bt2b	ACCCTCAGTGTAGTGACCCTTGGC	

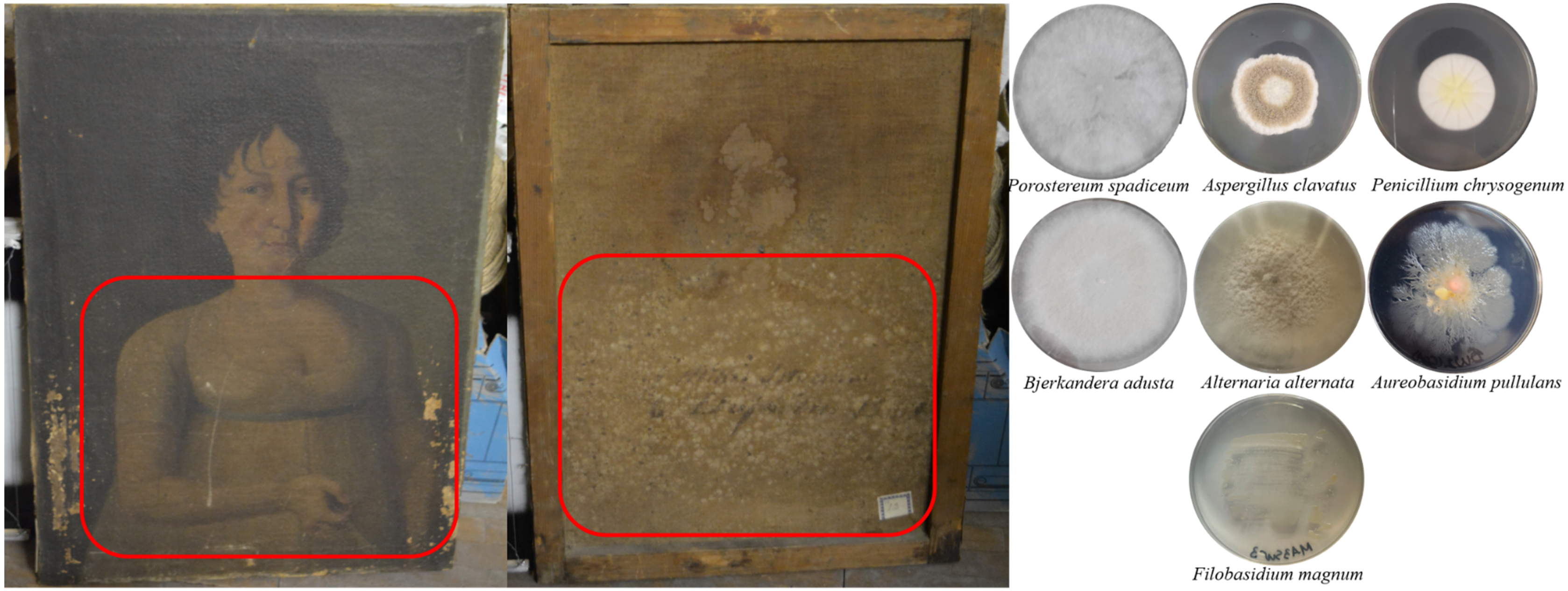

Figure S1. Oil on canvas painting MA3355, in the repository of The Art Museum, Cluj-Napoca, Romania, with red rectangles indicating the sampling areas and fungal isolates inhabiting the reverse of the support.

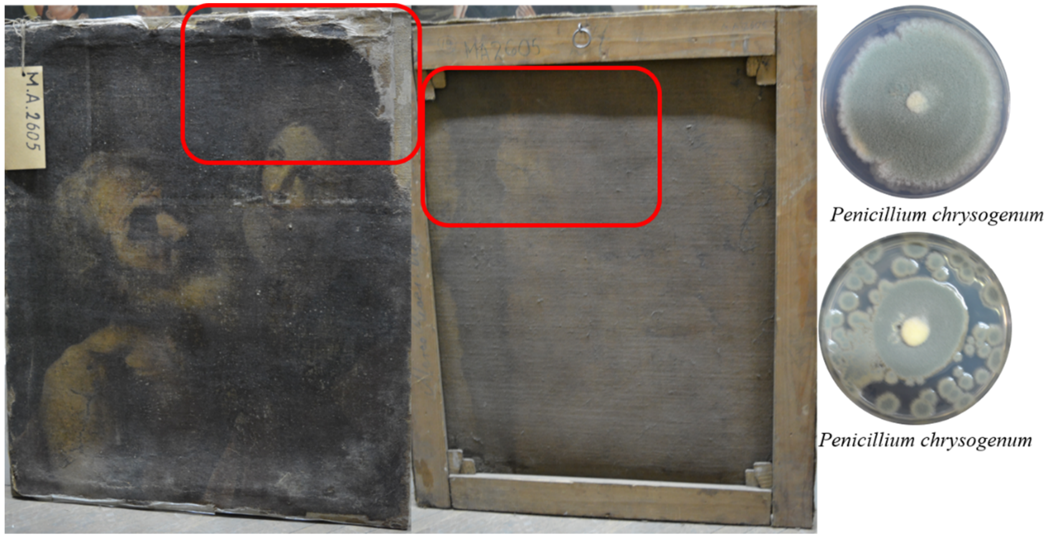

Figure S2. Oil on canvas painting MA2605, in the repository of The Art Museum, Cluj-Napoca, Romania, with red rectangles indicating the sampling areas and fungal isolates inhabiting the reverse of the support.

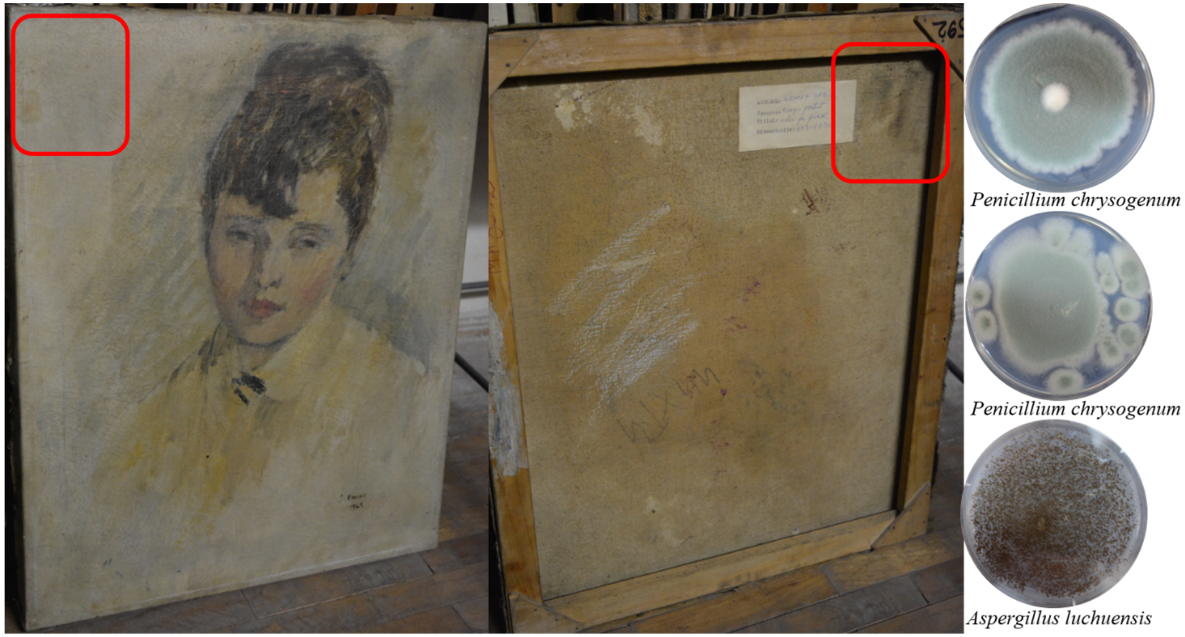

Figure S3. Oil on canvas painting MA8592, in the repository of The Art Museum, Cluj-Napoca, Romania, with red rectangles indicating the sampling areas and fungal isolates inhabiting the reverse of the support.

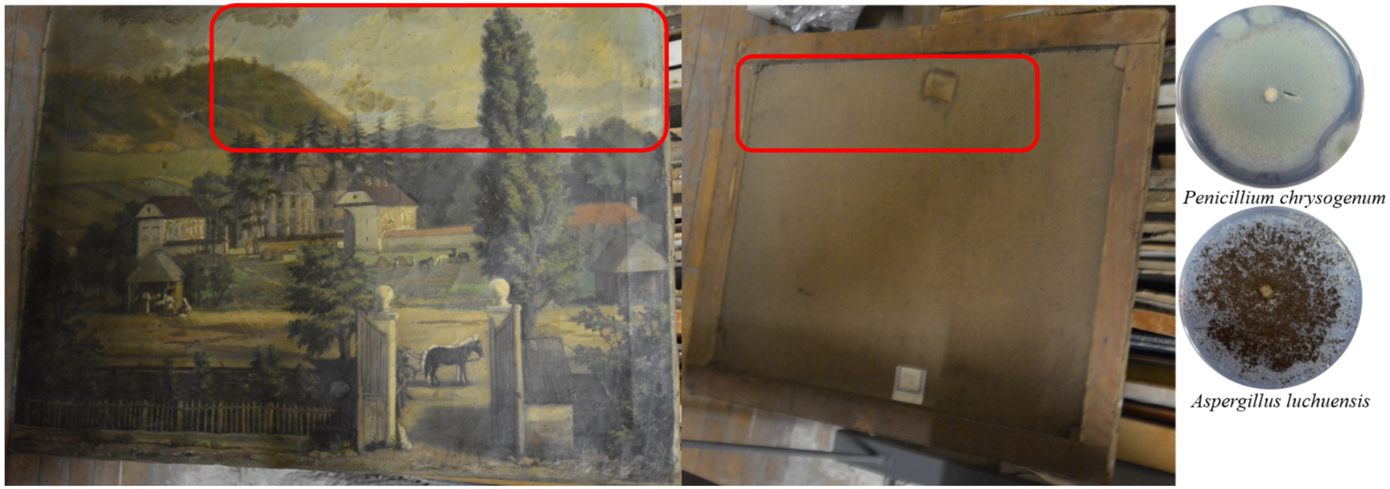

Figure S4. Oil on canvas painting MAFD261, in the repository of The Art Museum, Cluj-Napoca, Romania, with red rectangles indicating the sampling areas and fungal isolates inhabiting the reverse of the support.

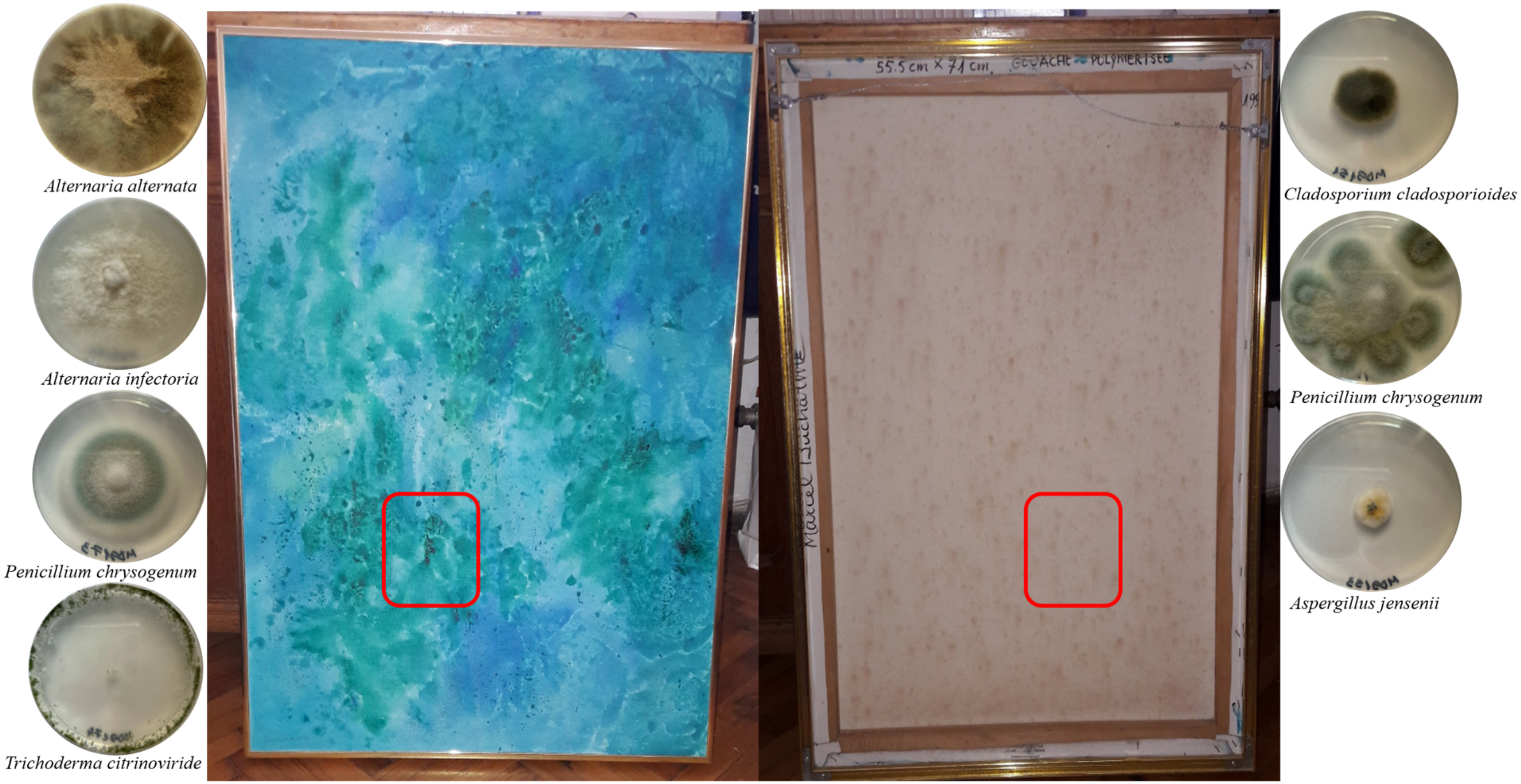

Figure S5. Gouache on canvas painting MD91, from a personal collection, Cluj-Napoca, Romania, with red rectangles indicating the sampling areas and fungal isolates inhabiting the observse (on the left) and the reverse (on the right) of the support.

Table S2. Identity of the isolated fungal species according to the blastn results of the amplified molecular markers.

ID	ITS			TEF			Bt2		
	Species	I (%)	Qc (%)	Species	I (%)	Qc (%)	Species	I (%)	Qc(%)
TeMATIC_A	***Porostereum spadiceum***	100	99	*Bjerkandera* sp.	100	77	*		

TeMATIC_B	*Aspergillus clavatus*	99.13	99	***Aspergillus clavatus***	99.83	99	*Aspergillus clavatus*	99.82	99
TeMATIC_C	***Penicillium chrysogenum***	99.59	100	*Penicillium chrysogenum*	98.96	99	*Penicillium chrysogenum*	99.84	99
TeMATIC_E	***Bjerkandera adusta***	100	99	*Bjerkandera* sp.	99.7	92	*		
TeMATIC_F	***Alternaria alternata***	99.26	99	*Alternaria maritima*	99	99	*		
MA3Sw2	*Aureobasidium pullulans*	97.2	96	***Aureobasidium pullulans***	99.35	99	*		
MA3Sw3	***Filobasidium magnum***	100	100	*			*		
IMAS1	***Penicillium chrysogenum***	100	100	*Penicillium rubens*	99.67	100	*Penicillium chrysogenum*	99.79	100
IMAS2	*Penicillium commune*	98.85	100	*Penicillium rubens*	99.67	99	***Penicillium chrysogenum***	99.57	100
IIMA3S1	*Penicillium* sp.	99.26	98	*Penicillium chrysogenum*	99.53	100	***Penicillium chrysogenum***	100	100
IIMA3S2	*Penicillium* sp.	99.44	100	*Penicillium* sp.	99.51	100	***Penicillium chrysogenum***	100	100
IIMA3S3	*Aspergillus* sp.	92.46	100	***Aspergillus luchuensis***	99.66	*99*	*Aspergillus* sp.	99.82	98
IIMA5S1	*Penicillium* sp.	98.13	100	*Penicillium chrysogenum*	99.36	99	***Penicillium chrysogenum***	100	99
IIMA5S2	*Aspergillus* sp.	98.9	98	***Aspergillus luchuensis***	99.38	100	*Aspergillus* sp.	99.82	98
MD91F1	***Alternaria alternata***	99.8	100	*Alternaria* sp.	99.84	99	*		
MD91F2	***Alternaria infectoria***	99.81	99	*Alternaria triticina*	99.65	100	*Alternaria dianthicola*	98.78	98
MD91F3	*Penicillium chrysogenum*	98.91	100	*Penicillium* sp.	99.19	98	***Penicillium chrysogenum***	99.78	98
MD91F4	***Trichoderma citrinoviride***	100	100	*Trichoderma citrinoviride*	99.84	99	*		
MD91S1	***Cladosporium cladosporioides***	100	100	*Cladosporium oxysporum*	99.37	98	*Aspergillus jensenii*	99.52	99
MD91S2	*Penicillium chrysogenum*	99.45	98	*Penicillium chrysogenum*	99.05	99	***Penicillium chrysogenum***	100	100
MD91S3	*Aspergillus versicolor*	95.63	99	*Aspergillus puulaauensis*	98.58	99	***Aspergillus jensenii***	99.3	100

- Sample not intended for amplification.

Based on the highest identity (I%) and query cover (Qc%) scores, the corresponding sequences of the investigated markers were selected for species identification (marked in bold and deposited at ncbi, under accession numbers detailed in the Materials and Methods section).
